# Conjugation Length Dependence of Intramolecular Singlet Fission in a Series of Regioregular Oligo 3-Alkyl(thienylene-vinylene)s

**DOI:** 10.1021/jacs.4c12877

**Published:** 2024-12-23

**Authors:** Daniel W. Polak, Iain Andrews, Enrico Salvadori, Andrew J. Musser, Alexander Auty, Dimitri Chekulaev, Julia A. Weinstein, Martin Heeney, Jenny Clark

**Affiliations:** † Department of Physics and Astronomy, University of Sheffield, Hounsfield Road, Sheffield S3 7RH, U.K.; ‡ Department of Chemistry and Centre for Processable Electronics, Imperial College London, White City Campus, London W12 0BZ, U.K.; § Department of Chemistry, NIS, University of Turin, Via P. Giuria 7, I10125 Torino, Italy; ∥ Department of Chemistry and Chemical Biology, Cornell University, 122 Baker Laboratory, Ithaca, New York 14853, United States; ⊥ Department of Chemistry, Dainton Building, The University of Sheffield, Brook Hill, Sheffield S3 7HF, United Kingdom of Great Britain and Northern Ireland

## Abstract

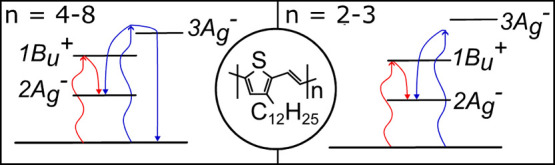

Activated intramolecular singlet fission is known to occur in the conjugated polymer polythienylene-vinylene (P3TV). Instead, efficient intersystem crossing has been observed in a short 3-alkyl(thienylene-vinylene) dimer. Here, we investigate a series of oligomers covering the conjugation length gap between the dimer and polymer. We confirm that the polymer and longer oligomers undergo activated intramolecular singlet fission, while the shorter oligomers with less than 3 units predominantly undergo efficient intersystem crossing. For the longer oligomers, the intermediate state of singlet fission is assigned to the 3A_g_^–^ covalent state of triplet pair character, as predicted by several computational studies. Our results point to potential pitfalls when assigning triplet production pathways solely with transient absorption spectroscopy.

## Introduction

Polyenes are a class of materials whose conjugated backbone is constructed of alternating single and double bonds.^1−6^ A defining characteristic of most polyenes is that their first excited state (2A_g_^–^, S_1_) has the same symmetry as the ground state.^5,6^ This means that one-photon transitions between the 2A_g_^–^ state and the ground state are symmetry forbidden, with absorption occurring instead to the second excited state (1B_u_^+^, S_2_).^5,6^

For many years it has been known that polyene covalent states, like the 2A_g_^–^ state, can be described as a pair of triplets.^7,8^ In 1987 Tavan and Schulten predicted that the triplets within this pair could separate and become isolated with a small energetic push, at a critical conjugation length.^9,10^ This implies the possibility of activated intramolecular singlet fission in long-chain polyenes, i.e., formation of two triplets from a single singlet state.^11,12^

Activated intramolecular singlet fission has since been observed in polyene-like polymers (polydiacetylene,^13−16^ P3TV^17^). For polydiacetylene the 2A_g_^–^ state was not observed before the formation of triplets due to singlet fission occurring entirely within the instrument response of these measurements.^13−16^ However, for P3TV, Musser and co-workers found that both the triplet pair and 2A_g_^–^ state form in parallel.^17^ These observations confirm that the 2A_g_^–^ state cannot be the parent triplet pair state of singlet fission in these systems.

Shorter oligomer variants of P3TV, the *n*TVs, have also been studied, due to their possible use in semiconductor devices.^18,19^ Apperloo et al. studied a series of *n*TV’s, discovering triplets throughout.^20^ However, due to the limited time resolution of the data presented in their study, they were unable to assign production pathways. Datko et al. investigated an *n*TV variant with two units observing rapid 100 ps intersystem crossing facilitated via a twisting motion^21^ with no signatures of singlet fission. As such, two questions remain open regarding P3TV/*n*TV singlet fission. What is the parent state of singlet fission?; and what is the critical conjugation length at which singlet fission becomes possible?

Herein, we measure a series of well-defined regioregular *n*TV oligomers running from 2 to 8 units using excitation dependent femtosecond transient absorption and time-resolved electron paramagnetic resonance spectroscopy. For the shorter oligomers we observe efficient intersystem crossing, most likely progressing via a large-scale geometric reorganization. At 4 units we observe a distinct split, with the 4–8 unit oligomers undergoing a form of activated intramolecular singlet fission. By comparison to recent computation results, we suggest the most likely candidate for the triplet pair state is the 3A_g_^–^ state. Finally, we discuss how the presented data points to potential pitfalls when assigning triplet production pathways using transient absorption spectroscopy dynamics alone.

## Results

**Figure 1:**
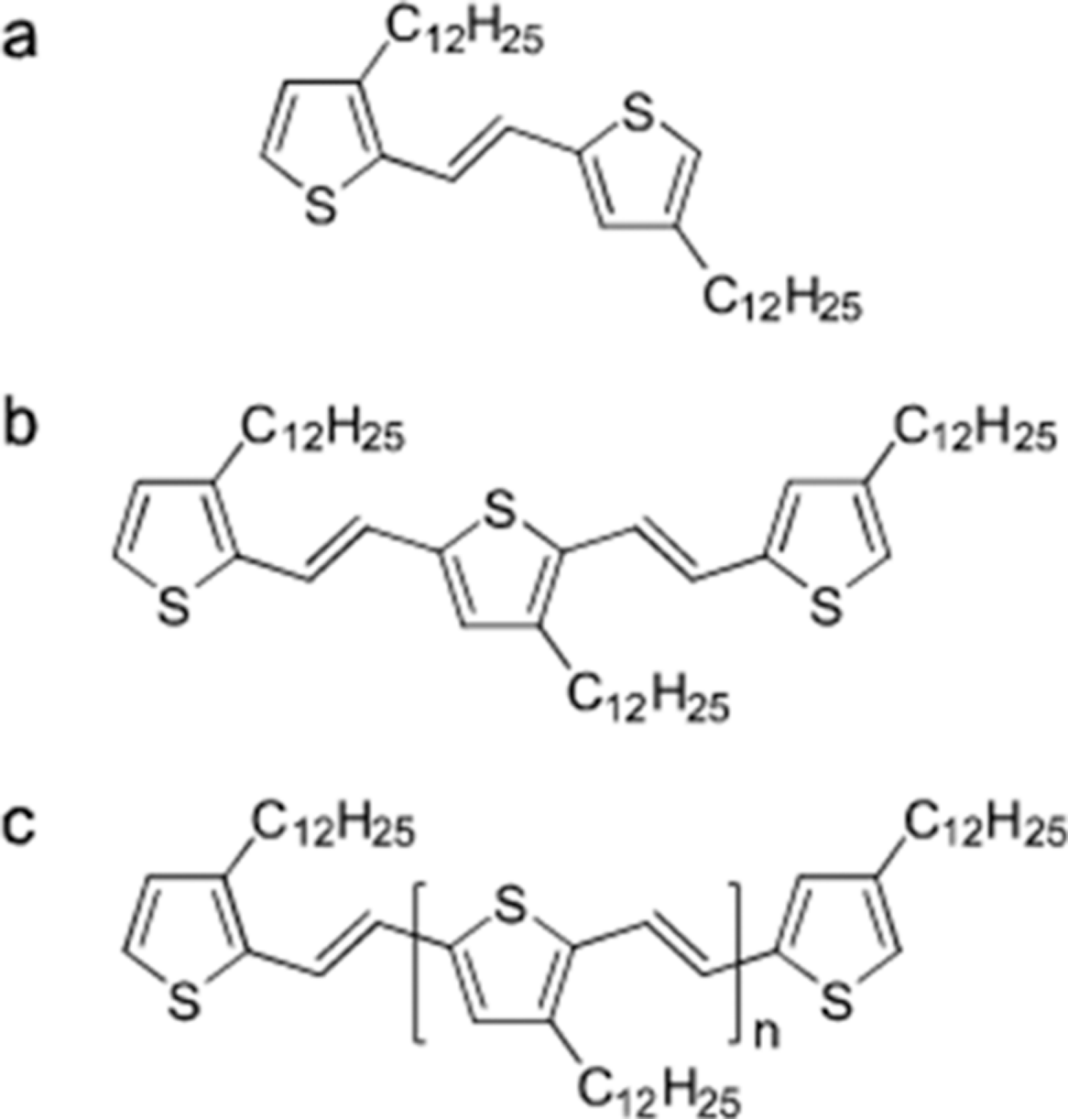
Molecular Structure of the *n*TV oligomers. Molecular Structure of the *n*TV dimer (a), trimer (b), and generic structure of the longer oligomers (c), where n is the number of repeat units with 2 for the tetramer, 3 for the pentamer etc.

Figure 1 shows the molecular structure of the oligomer series discussed below. For steady state spectra and details of the synthetic method we refer the reader to ref (22). In a previous study, it was shown that at concentrations used in the present study, oligomers in toluene remain isolated with no sign of aggregation.^22^ To rule out intermolecular interactions leading to singlet fission in any solvent used here, in Figure S1, we compare transient absorption data obtained in pure ethanol to data obtained in toluene. As we observe no change in the transient data between ethanol and toluene, we confirm the oligomers remain isolated. Here we begin with time-resolved studies of each oligomer, starting with the shortest oligomer, the dimer.

### Dimer

**Figure 2:**
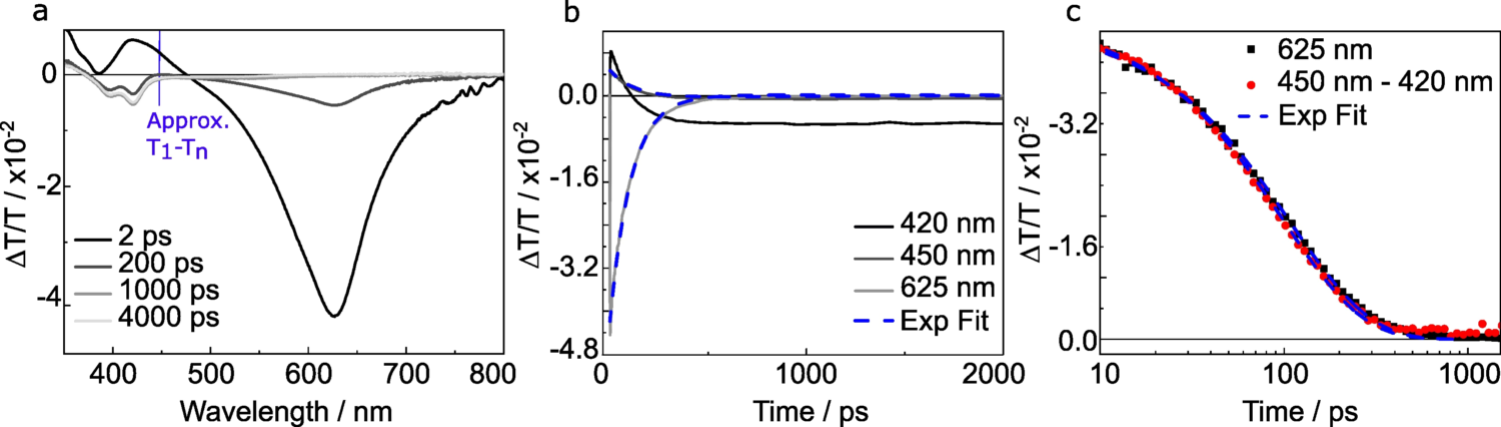
Transient absorption spectroscopy of the *n*TV dimer. Transient absorption spectroscopy in 180 μMol toluene solution following 320 nm excitation, showing spectra averaged over 2–3 ps, 200–300 ps, 1–2 ns, 4–6 ns (a) and kinetics averaged over a 10 nm window centered on the wavelengths shown in the legend (b). An estimate of the dimer triplet energy is extrapolated from ref^20^ (c) Comparison of the 625 nm kinetic (reproduced from panel b) to the scaled difference kinetic described in the text.

In Figure 2 we present transient absorption spectra and kinetics for the *n*TV dimer in toluene solution excited at 320 nm. At a 2 ps pump–probe delay, the spectrum is dominated by two features, a positive peak at 400–450 nm which has a line shape similar to that of the dimer emission, and a negative excited state absorption (ESA) peak at 625 nm. As both features rise instantaneously and decay together, we assign both to the absorbing state, in agreement with measurements of a similar *n*TV dimer.^21^

The decay of both peaks can be described by a biexponential function with decay constants: 115 ± 5 ps and 1.9 ± 0.2 ns. During the decay of both features we observe a new ESA form at 420 nm underneath the stimulated emission. After 2 ns we find no further evolution with the 420 nm peak showing minimal decay within the measurement window, indicating a ≫2 ns lifetime. To assign this state, in Figure 2a we show the expected T_1_–T*_n_* transition energy for the dimer extrapolated from the T_1_–T*_n_* energy versus conjugation length plot by Apperloo et al.^20^ We find an agreement between this energy and position of the ESA feature which, combined with the long lifetime, suggests the feature is related to triplets.

While we cannot resolve the rise of this feature directly, we do observe a faster decay at 420 nm than at 450 nm (Figure 2b). This is caused by the combination of the decay of the stimulated emission and the rise of the 420 nm ESA. To pull these apart we subtract the 450 nm kinetic (SE decay) from the 420 nm kinetic, to attempt to isolate the rise of the peak. Figure 2c presents a scaled comparison of the rise of the recovered 420 nm feature with the decay of the 625 nm singlet feature. We find a similarity between the two, suggesting there is an interconversion of the absorbing state and the triplet-like state, quenching the stimulated emission.

In their study of P3TV, Musser and co-workers demonstrated that intramolecular singlet fission only occurs when activated by excess energy excitation,^17^ as previously observed in polydiacetylene.^13−16^ To rule out excess energy activated singlet fission here, we measure the dimer at 320 and 370 nm excitation (Figure S2). We find no excitation dependence, ruling out activated singlet fission occurring through the same mechanism as the polymer. Overall, our measurements are consistent with 100 ps intersystem crossing as previously observed for a similar *n*TV dimer.^21^ We now move to the next oligomer in the series: the trimer.

### Trimer

**Figure 3:**
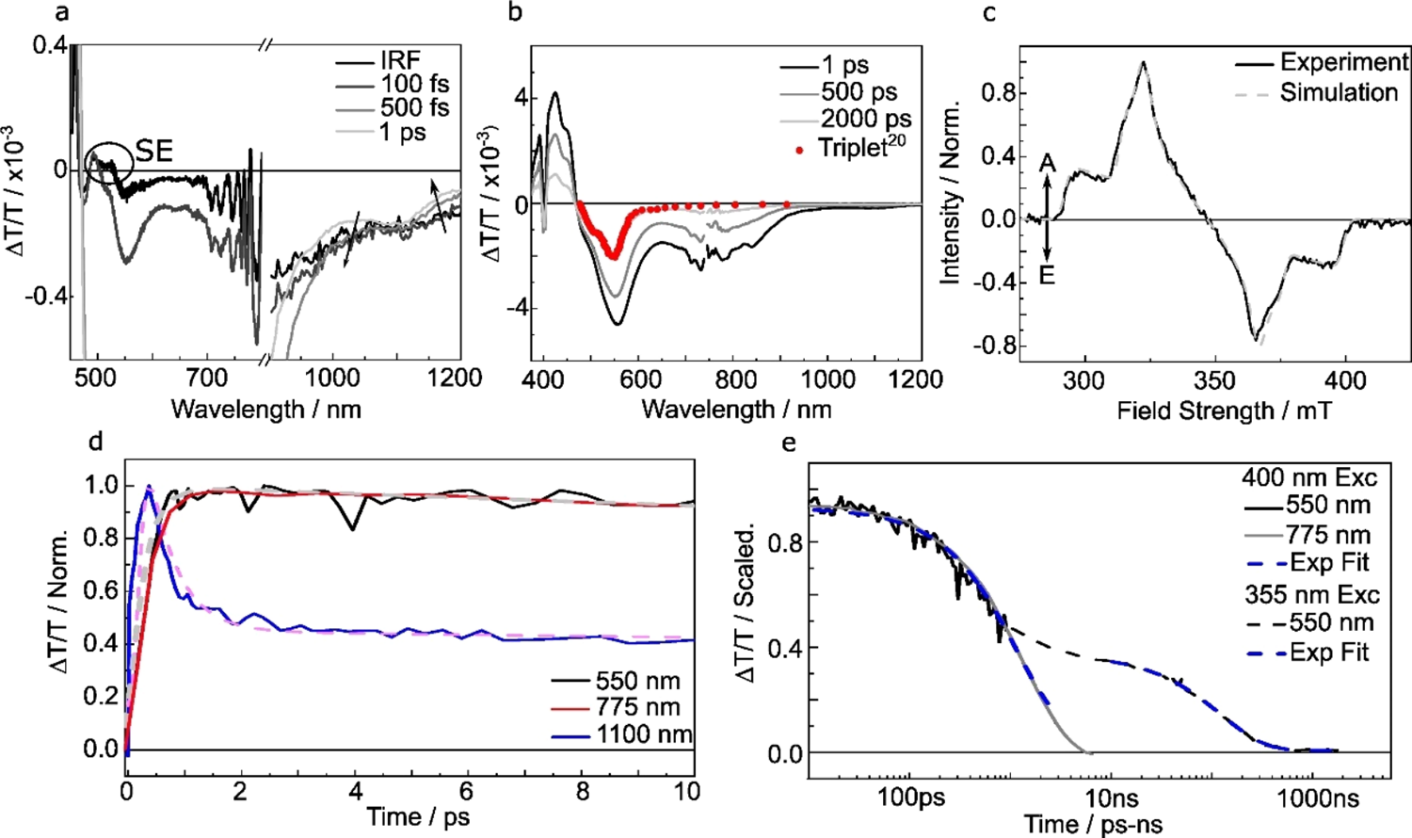
Transient absorption spectroscopy of the *n*TV trimer. (a, b) Transient absorption spectra of the trimer excited at 400 nm in 110 μMol toluene solution, averaged for 100 fs (a) and 10 ps (b) around the delays shown in the legend. In panel b we include the sensitized triplet spectrum from Apperloo et al. reproduced from ref (20). Copyright 2002 American Chemical Society. (c) EPR measurements conducted at 9.6 GHz after 410 nm excitation with relative simulation (A = enhanced absorption, E = emission). Samples were prepared at 77 K in a mixture of ethanol:toluene:diethyl ether in a 1:1:2 ratio. The solution was degassed to remove oxygen. For further details, see the materials section. (d, e) Transient absorption kinetics averaged over a 10 nm window around the wavelengths displayed in the legend (excitation at 400 nm). We also include a ns-TA kinetic at the triplet peak excited at 355 nm (0.5 mW power). Note the overlap of the ps-TA and ns-TA kinetics, allowing accurate scaling. In panel d exponential fits are indicated with dashed lines, fit parameters are listed in the Supporting Information.

In Figure 3 we present transient absorption spectra taken after 400 nm excitation of the *n*TV trimer in a toluene solution. Initially, we observed two spectral features, best viewed at a pump–probe delay of 50 fs (within the instrument response, Figure 3a). We observe a small, stimulated emission (SE) feature at 500–530 nm, and the tail of an ESA in the near-infrared (NIR) at 1100 nm. This is similar to previous measurements of astaxanthin^23^ (a polyene) and previous studies of *n*TV systems.^22,24^ In these studies, the authors observe a strong ESA in the IR region from the 1B_u_^+^ (absorbing) state.^23,24^ By comparison, we assign the stimulated emission and the NIR ESA tail to the absorbing state of B_u_ symmetry.

Over the same time scale as the loss in the features relating to the 1B_u_^+^ state, we observe the rise of two features at 550 and 775 nm covering the whole measurement window (Figure 3b). Note the long-lived offset in the 1100 nm kinetic is due to overlap with the tail of the 775 nm feature. Both 550 and 775 nm ESA features can be fit with a single rise constant just outside the IRF of 280 ± 50 fs and a decay constant of 1.3 ± 0.2 ns (Figure 3e). For more details of the kinetic fits, we refer the reader to section 2 of the Supporting Information. As both features have identical early time kinetics (Figure 3e) and rise with the same time constant as the decay of the 1B_u_^+^ state we suggest they both represent the same state formed from the 1B_u_^+^ state.

Interestingly, the 550 nm feature appears very similar to the sensitized triplet spectrum measured by Apperloo et al. (red squares, Figure 2b) but red-shifted by 6 meV.^20^ This is qualitatively similar to previous observations of triplet pair states in which the ESA matches an isolated triplet spectrum but shifted due to binding between the triplets in the pair.^25−27^

The assignment of the state as having a triplet pair character also potentially explains the additional peak at 775 nm. Mazumdar and co-workers studied ^1^(TT) states theoretically for a series of acene dimers calculating the excited state absorption spectrum.^28,29^ They find that in addition to a triplet character transition in the visible, there are also smaller peaks related to 1e–1h charge transfer configurations.^28,29^ These observations qualitatively match the ESA features measured here for the trimer. Together, this suggests the features can be collectively assigned to a state formed from the absorbing state with triplet pair like features. While this implies singlet fission, recent measurements of polyenes have shown that covalent states also have ESA features which match triplet pair states formed via more exotic processes.^25,27^ To rule out activated singlet fission as in the polymer, we measure excitation dependent transient absorption (Figure S3). We find no excitation dependence confirming the trimer does not undergo activated singlet fission. As a result, in agreement with previous measurements of polyene systems,^6,17,23,24^ including the trimer itself,^22^ we assign these features to the well-known 2A_g_^–^ state of polyene systems formed via internal conversion.

However, in other previously measured polyene systems, the covalent 2A_g_^–^ state is observed to have a lifetime on the order of picoseconds^6,17,23,24^ while here the associated features appear to have considerable population past the end of our measurement window (2 ns). To investigate, we measure the trimer on a transient absorption setup with a microsecond delay range. A characteristic spectrum at a 1 μs delay from this set of measurements is shown in the Supporting Information (Figure S4). While we no longer clearly observe the peak at 775 nm, the 550 nm feature lives far longer in the microsecond domain.

The 550 nm kinetics from these measurements has been spliced into Figure 3e. We observe a monoexponential decay with a 125 ± 20 ns decay constant. As our measurements were conducted in the presence of oxygen we expect an isolated triplet lifetime on the order of 100 ns.^21^ This is due to the well documented quenching of triplets by oxygen.^30^ Further, at post 2 ns pump–probe delays, the 550 nm feature is slightly blue-shifted, now matching the spectral shape ***and position*** of isolated triplets. These observations suggest that at longer delays isolated triplets form, most likely from the state with triplet pair like features (2A_g_^–^). While we ruled out activated singlet fission, the above observation leads to a complicated distinction. The 2A_g_^–^ state is a singlet state, but it can also be described as having triplet pair character. If isolated triplets form from this state, is this considered a form of singlet fission or intersystem crossing? Transient absorption spectroscopy is unable to resolve this issue; however, time-resolved electron paramagnetic resonance (TR-EPR) experiments can.

TR-EPR is a technique that is sensitive to paramagnetic intermediates such as triplets and charges.^31−34^ The time resolution of our TR-EPR experiment is on the order of 100–150 ns so only allows detection of species that are long-lived on the time scale of fs-transient absorption experiments.^31,32^ As a result, TR-EPR preferentially probes the long-lived triplets and not the bound triplet pair state. Importantly the polarization of the spectrum collected provides valuable information about the mechanism responsible for the formation of the triplets.

For example, it has been shown that singlet fission preferentially populates the *M*_s_ = 0 triplet sublevel.^35^ This leads to a distinctive polarization pattern of AEEAAE (*A* = enhanced absorption, *E* = emission) as seen in pentacene and tetracene.^31,32^ However, for intersystem crossing we expect no preferential population of the *M*_s_ = 0 level leading to a EEEAAA^36,37^ or a AAAEEE^36,38,39^ polarization pattern. In Figure 2e we show the TR-EPR spectrum of the trimer along with a simulation with parameters *D* = −1500 MHz, *E* = 90 MHz and triplet sublevel populations P*_X_*/P*_Y_*/P*_Z_* = 0.39:0.44:0.17. We see a clear polarization pattern AAAEEE, which along with the fit parameters implies a preferential population of the *X* and *Y* zero-field triplet sublevels.

This polarization pattern allows us to exclude that the measured spectrum results directly from a singlet fission event. Such a pattern has been suggested to point to the involvement of out-of-plane spin–orbit coupling.^38^ This would be consistent with the model presented by Datko et al. for an *n*TV dimer.^21^ Together this confirms that for the trimer, we observe intersystem crossing originating from the 2A_g_^–^ state. Interestingly, this state which has significant triplet pair character, appears to undergo intersystem crossing with no variation from other singlet character states. This result is consistent with previous observations of intersystem crossing in carotenoids, in which triplet formation occurs with expected trends from the 2A_g_^–^ state.^40^ This observation is also expected from an energetic argument when considering that the triplet pair is contained on a single molecule. Estimates for the splitting of singlet character triplet pair, and the higher spin triplet pair states for intramolecular systems are well above thermal energy at room temperature.^41^ As such, for strongly bound systems such as these, the quintet and triplet character triplet pair states are inaccessible, producing a purely singlet character state.

These results once again point to a potential pitfall in identifying singlet fission systems using only transient absorption dynamics. Using transient absorption spectroscopy, we have observed the formation of a state with triplet pair like features which convert to long-lived isolated triplets, a hallmark of singlet fission systems. However, via TR-EPR and excitation energy dependent measurements, we have ruled out any form of singlet fission in the trimer. With the trimer assigned to the dimer model, we move to investigate the longer chain oligomers below.

### Long Chain Oligomers

**Figure 4:**
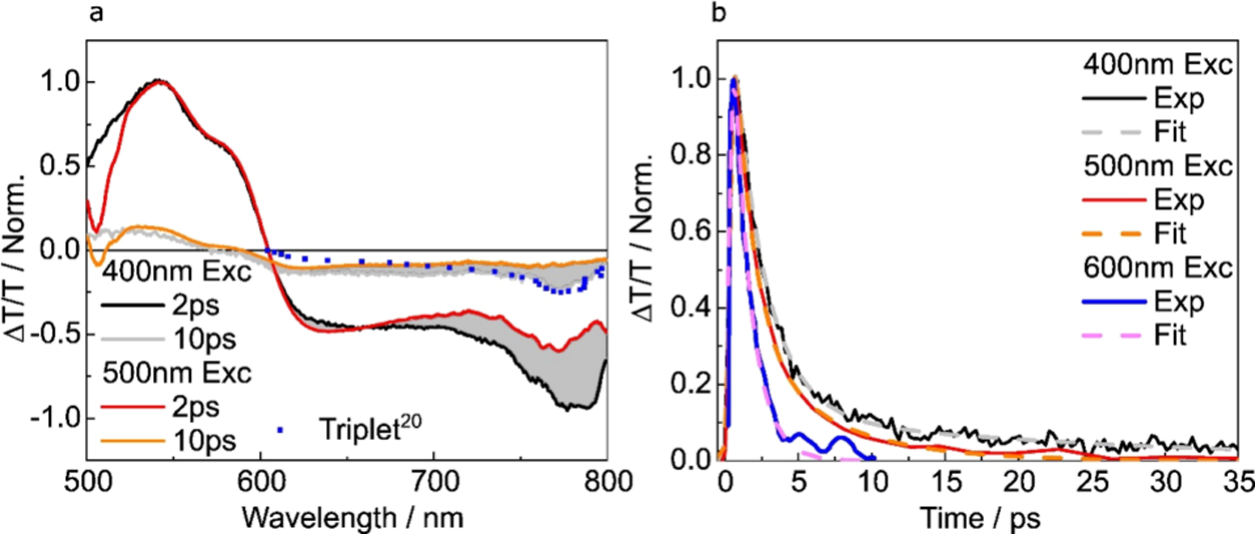
Excitation energy dependent transient absorption of the *n*TV octamer: (a) Transient absorption spectra of the octamer excited at 400 and 500 nm in 50 μMol toluene solution. Spectral slices are averaged over 1 ps windows, starting at the delays marked in the legend. The sensitized triplet spectra taken from Apperloo et al. is also included, blue-shifted by 180 meV, reproduced from ref (20). Copyright 2002 American Chemical Society. The 500 nm spectra have been scaled so that the 0–0 peak of the ground state bleach matches the 400 nm excitation data. (b) Transient absorption kinetics of the octamer taken at the peak of the triplet feature (775–785 nm) for 400, 500, and 600 nm excitation. Exponential fits are also included as dashed lines.

In Figure 4 we present transient absorption spectra and kinetics for the *n*TV octamer in toluene solution excited at 400, 500, and 600 nm. 600 nm excitation spectra have been omitted due to overlap of features with pump scatter, making it impossible to scale accurately for comparison. Oligomers with 4–7 units have similar dynamics to those in Figure 4 and are presented in the Supporting Information (Figure S5). From the earliest pump–probe delays, we observe no stimulated emission, with a single positive feature that can be assigned to the ground state bleach due to its spectral shape and position for all long oligomers. The lack of a stimulated emission peak suggests that the 1B_u_^+^–2A_g_^–^ interconversion observed in the other systems must be much shorter than our instrument response (∼300 fs).

In addition to the ground state bleach, we also observe a broad ESA that covers the measurement window for all systems. In Figure 3b we present kinetics associated with the ESA taken after 400, 500, and 600 nm excitation. Fitting to the 600 nm excitation kinetics yields a single decay constant of 1.4 ± 0.3 ps, which matches the recovery time of the ground state bleach. Equivalent kinetics and fits are shown for the 4–7-unit oligomers in the Supporting Information (Figure S5). By comparison to the previously studied systems, we assign the short-lived feature to the 2A_g_^–^ state.^17,22^

Fitting to the 400 and 500 nm excitation kinetics yields two exponential components with no additional rise outside the IRF. The first matches the 2A_g_^–^ state lifetime (1.4 ps), and the second is 24 ± 5 ps, with increasing amplitude going from 500 to 400 nm excitation. As we observe no new slow rising component, and no change in the first decay component, both states must be formed in parallel within the IRF rather than sequentially.

Comparing transient spectra after 400 and 500 nm excitation displays a distinctly different spectral shape, with an enhancement in the 600–800 nm region of the spectrum at higher energy excitation as seen previously for P3TV.^17^ In Figure 4a, we also include a sensitized triplet spectra measured by Apperloo et al.^20^ blue-shifted by 180 meV. There is a clear similarity between the enhancement at high energy excitation (indicated with gray shading) and the sensitized triplet spectrum.

Similar shifts have been observed empirically previously for a variety of singlet fission systems in which it was suggested that the shift is quantitatively linked to the binding energy between the triplets in the pair state.^25−27^ Such a suggestion assumes that the triplet pair excited state absorption occurs to a high energy triplet state. In contrast, recent computational studies of triplet pair excited state absorptions suggest the transition instead occurs from the known charge transfer component of the triplet pair state.^42,43^ Such an interpretation does not account for the experimentally observed correlation of this shift with binding energy, pointing to the need for further studies on this topic.

Equivalent assignments, fits, and comparison to published sensitized triplet spectra can be made for other oligomers shown in the Supporting Information (Figure S5). Overall, for the 4–8 unit oligomers our observations are consistent with those presented previously for P3TV in which activated intramolecular singlet fission occurs in parallel with formation of the 2A_g_^–^ state.^17^

A question remains as to what triplet pair state the activated singlet fission is occurring from. Valentine et al.^44^ among others^45^ have published computational studies investigating the higher lying covalent states in polyenes for their involvement in singlet fission. The authors found that the ladder of covalent states (2A_g_^–^, 1B_u_^–^, 3A_g_^–^) are all potential singlet fission intermediates with significant triplet pair character. Recently, for intermolecular singlet fission in a benzodipyrrolidone, Wang and co-workers observed experimental and theoretical evidence for the singlet fission intermediate state being the 3A_g_^–^ state.^46^ To consider whether this could also be true for the *n*TV oligomers, we examine the energetics of the triplet pair as a function of chain length.

For the longer chain *n*TV’s we observe singlet fission only after excitation above the 1B_u_^+^ state minimum, implying an energetic barrier and requiring the triplet pair state to sit above the 1B_u_^+^ state with a small energetic gap. While for shorter oligomers, the triplet pair state must lie much higher in energy and so be inaccessible, and unable to take part in singlet fission at all.^42^ For a bare polyene chain Tavan and Schulten used varied methods to calculate the energies of several covalent states.^9,10^ The 3A_g_^–^ state was predicted to lie above the absorbing state, with an energy gap increasing from 0.1 eV to over 1 eV at shorter conjugation lengths. As such, the tentative assignment of 3A_g_^–^ as the triplet pair state is consistent with the measurements presented here.

Such an assignment is consistent with several studies that have suggested accessing higher lying states could improve the performance of singlet fission materials, termed antikasha singlet fission.^47,48^ By accessing higher lying singlet states, it is proposed that new routes to singlet fission can be opened, a process that has been observed experimentally,^49^ and is similar to what we observe in the longer oligomers.

## Discussion

Here we discuss the definition of intramolecular singlet fission. Singlet fission is defined as a singlet state converting to two triplets.^11^ However, in acene literature due to the prominence and importance of intermediate states, a new terminology has become more common.^27^ In this new language, singlet fission is sometimes defined as the process of a singlet state converting to a singlet character triplet pair state. If we apply these descriptions of singlet fission to the *n*TV’s we arrive at some complicated results.

Using the above definition, the longest oligomers undergo intramolecular singlet fission, from the absorbing state to two states with triplet-like transitions (2A_g_^–^, 3A_g_^–^), where neither separates to free triplets. Instead, both states decay in parallel with a similar decay rate to singlet states in polyenes. Combined with the triplet-like spectral line shapes, and the triplet pair character^9,10^ of covalent singlet states regardless of the involvement of singlet fission, this makes 1B_u_^+^–2A_g_^–^ or 1B_u_^+^–3A_g_^–^ internal conversion and intramolecular singlet fission without triplet separation essentially equivalent, when viewed through transient absorption dynamics alone. The processes of singlet fission and internal conversion are only distinguished via the characteristic excitation energy dependence of singlet fission previously characterized.

However, for P3TV upon incorporating into a film with a triplet acceptor, the triplets separate and are even able to undergo charge formation.^17^ This implies that there are differences between singlet fission and internal conversion to covalent states with significant triplet pair character in polyenes. Additionally, for the trimer we observe formation of isolated triplets from a state with triplet-like features which implies singlet fission; however, on investigation we find intersystem crossing is a more apt description when observing the triplets via TR-EPR experiments and through the lack of an excitation energy dependence in its transient features.

Together these issues highlight the potential pitfalls when assigning triplet production pathways when solely using transient absorption dynamics. Further, our results indicate a need for more studies of the intermediate states in various systems to arrive at a consistent definition of the singlet fission process without the ambiguities created by the new nomenclature.

## Conclusions

The results presented here form a unified description of triplet production mechanisms in *n*TV materials. For the dimer and trimer, we find a different behavior compared with the longer oligomers. In these short oligomers, intersystem crossing to form isolated long-lived (μs) triplets is observed. This combined with evidence of large geometric reorganization for these molecules agrees with the previous study by Datko et al.^21^ However, in the longer oligomers (>4 units) we find evidence for parallel formation of the symmetry forbidden 2A_g_^–^ state and activated intramolecular singlet fission, as in the polymer.^17^ By comparison to previous work, we suggest that this intermediate state can be assigned to the higher energy state of covalent character, the 3A_g_^–^ state. Due to the similarity in spectral line shapes and lifetimes of internal conversion and intramolecular singlet fission, our measurements point to potential pitfalls when assigning singlet fission pathways using transient absorption dynamics alone.

## Experimental Section

### Sample Preparation

*n*TV molecules were synthesized by a Horner-Wadsworth-Emmons reaction, a full description of the synthetic process is described in ref (22). All solvents and chemicals involved in the synthesis were purchased from Sigma-Aldrich, VWR, Fischer Scientific or Fluorochem and used as received. The solutions were prepared in toluene at an OD of ∼0.4–0.6 (specific concentrations given in Figure captions) in a 1 mm path length cuvette unless stated otherwise in the Figures.

### Transient Absorption Spectroscopy

Transient absorption spectroscopy data was collected on a modified commercial Helios transient absorption spectrometer (HE-VIS-NIR-3200, Ultrafast Systems) as described previously.^22^ Briefly, the Helios system was driven by 800 nm pulses (∼40 fs, 10 kHz, 1.2 mJ) from a Spitfire Ace PA-40 (Spectra-Physics). The output was split into the pump and probe lines. The narrowband 400 nm visible pump pulses were produced by doubling the output using a β-barium borate (BBO) crystal, while the probe was produced using a Sapphire crystal (visible region) or an Yttrium aluminum garnet crystal (near-IR region).

### Time-Resolved Electron Paramagnetic Resonance Spectroscopy

Time-Resolved EPR (TR-EPR) experiments at the X-band frequency (∼9.6 GHz) were recorded on a Bruker E580 pulsed EPR spectrometer equipped with a Bruker ER4118X-MD5 dielectric resonator. TR-EPR spectra were recorded in direct detection mode without magnetic field modulation; therefore, they show characteristic enhanced absorptive (*A*) and emissive (*E*) features, as indicated in the reported spectra. A Surelite broadband OPO system within the operating range 410–680 nm, pumped by a Surelite I-20 Q-switched Nd:YAG laser with second and third harmonic generators (10 Hz, pulse length of 5 ns) was used to achieve a pulsed laser excitation at an appropriate wavelength optimized on the signal intensity, with the energy at the sample approximately 5 mJ per pulse. A cryogen-free cryostat from Cryogenic ltd. and a Lake Shore temperature controller (model 350) were used to cool the sample and maintain the temperature at 77 K. EPR samples were prepared at a concentration of 0.05 mg mL^–1^ in a mixture of ethanol:toluene:diethyl ether in a 1:1:2 ratio (v/v). The solvent mixture was used to allow formation of an optically clear glass at low temperate. TR-EPR spectra were simulated using the EasySpin toolbox^50^ in MATLAB to extract ZFS parameters −*D* and *E*– and sublevel populations (P*_x_*, P*_y_*, P*_z_*). The simulation of the *n*TV timer considered the ZFS parameter *D* negative, as expected for polyenes, whereas *E* was assumed as positive. An isotropic *g* value equal to the free electron *g* value (*g*_x_ = *g*_y_ = *g*_z_ = 2.0023) was used in the simulations.

## Supporting Information Available

Excitation wavelength dependent transient absorption spectroscopy of oligomers and details of the kinetic fitting procedures (PDF)

## Notes

Data from the manuscript can be accessed from the University of Sheffield data repository: https://doi.org/10.15131/shef.data.28067198.v1.
