# Supplementary material for: Conjugation Length Dependence of Intramolecular Singlet Fission in a Series of Regioregular Oligo 3-Alkyl(thienylene-vinylene)s
Source: J Am Chem Soc. 2024 Dec 23;147(1):662–8. doi: 10.1021/jacs.4c12877 (PMC11726551; doi:10.1021/jacs.4c12877)
Supplement: Supplementary file 1 — ja4c12877_si_001.pdf [file ja4c12877_si_001.pdf]

Supporting information: Conjugation Length Dependence of Intramolecular Singlet Fission in a Series of regioregular oligo 3-alkyl(thienylene-vinylene)s.

Daniel W. Polak,[1] Iain Andrews,[2] Enrico Salvadori,[3] Andrew J. Musser,[4] Alexander Auty,[5] Dimitri Chekulaev ,[5] Julia A.Weinstein,[5] Martin Heeney,[2] Jenny Clark[1]

[1] Department of Physics and Astronomy, University of Sheffield, Hounsfield Road, Sheffield S3 7RH, UK

[2] Department of Chemistry and Centre for Processable Electronics, Imperial College London, White City Campus, London, W12 0BZ UK

[3] Department of Chemistry, NIS, University of Turin, Via P. Giuria 7, I10125 Torino, Italy

[4] Department of Chemistry and Chemical Biology, 122 Baker Laboratory, Ithaca, New York 14853

[5] Department of Chemistry, Dainton Building, The University of Sheffield, Brook Hill, Sheffield S3 7HF

*Author for correspondence: jenny.clark@sheffield.ac.uk

Contents

1/ Transient Absorption Spectroscopy 2

2/ Kinetic Fitting 5

1/ Transient Absorption Spectroscopy

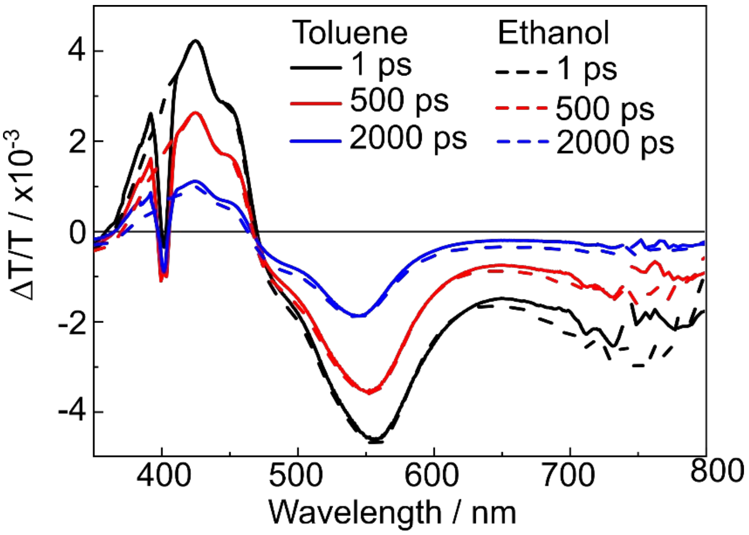

Figure S1: Solvent dependant transient absorption spectroscopy of *n*TV trimer. (a) Transient absorption spectra of the trimer in 110 µMol toluene and ethanol solution excited at 400 nm, averaged over time frames given in the legend (1 mW power).

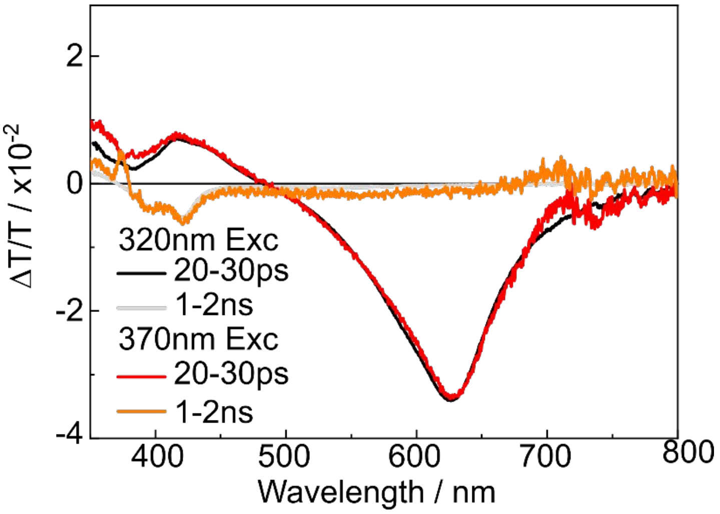

Figure S2: Excitation energy dependant transient absorption spectroscopy of nTV dimer. (a) Transient absorption spectra of the dimer in 180 µMol toluene solution excited at 320 nm and 370 nm, averaged over time frames given in the legend (1 mW power). We find no effect of excess energy excitation on the spectrum of the dimer.

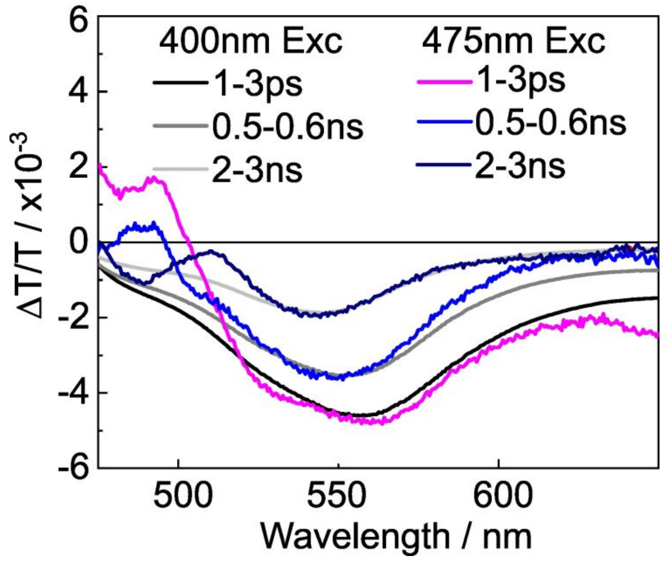

Figure S3: Excitation energy dependant transient absorption of the nTV trimer. Transient absorption spectra of the trimer excited at 475 nm and 400 nm in 110 μMol toluene solution (1 mW power). Spectral slices are averaged over times marked in the legend.

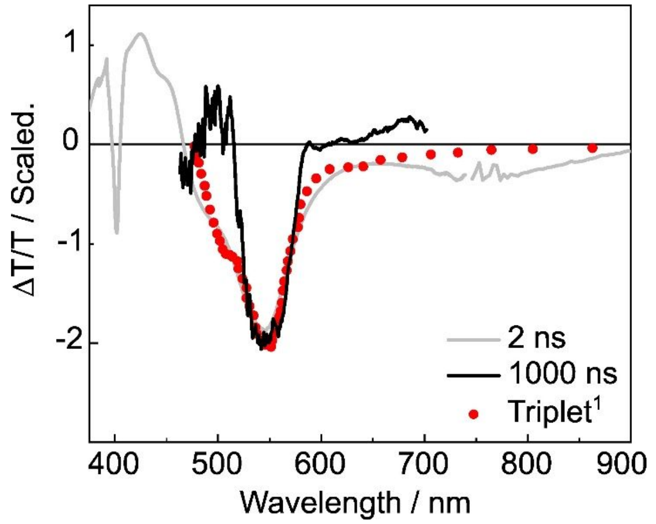

Figure S4: Microsecond transient absorption spectroscopy of nTV trimer. Transient absorption spectrum after 355 nm excitation (0.5 mW power) at a 1 μs pump probe delay (with Savitzky–Golay filter applied), scaled and compared to a spectrum taken after 400 nm excitation (1 mW power) at a 2 ns pump probe delay. Also included is the sensitised triplet spectrum for the trimer taken from Apperloo et al. reproduced from Ref [1]. Copyright 2002 American Chemical Society.

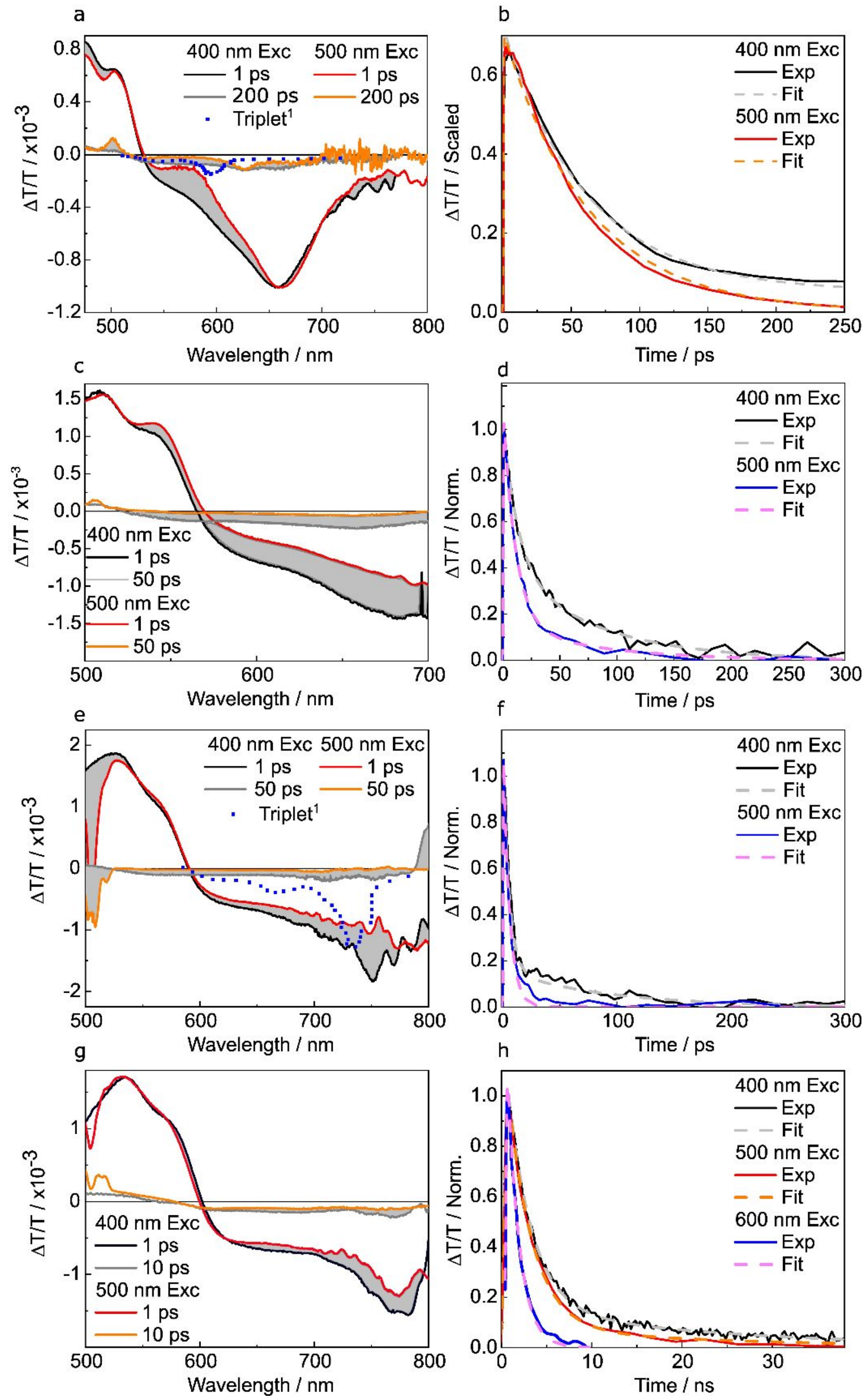

Figure S5: Excitation energy dependent transient absorption of 4-7 unit nTVs. Transient absorption spectra of the tetramer (80 μMol, a) pentamer (72 μMol, c), hexamer (64 μMol, e), and heptamer (60 μMol, g) excited at 400 nm and 500 nm. Spectral slices are averaged over 1 ps windows starting at delays marked in the legend. We also include the sensitised triplet spectra obtained by Apperloo *et al.*, blue-shifted by 140 meV (tetramer) and 120 meV (hexamer), reproduced from Ref [1]. Copyright 2002 American Chemical Society. The 500 nm spectra have been scaled so that the 0-0 peak of the ground state bleach matches the 400 nm excitation data. Transient absorption kinetics of the tetramer (600 nm) (b), pentamer (600 nm) (d), hexamer (725

nm) (f), and heptamer (775 nm) (h) averaged over a 10 nm window. Dashed lines show mono/bi exponential fits, fit parameters are detailed in the Table 1.

2/ Kinetic Fitting

All kinetics where fit with a gaussian describing the IRF convoluted with exponential rise and decay functions. The gaussian is locked to the pulse length of 360±10 fs which was identified via fitting the rise of the GSB features of several systems and averaging the result. Time zero is locked to 0 fs after chirp correction, in all fits below. For 4-8 unit oligomers the kinetics were averaged over a 10 nm window at the peak of their ESA. For trimer and dimer the wavelength the kinetic was averaged over is given in the table.

Table 1: Kinetic fitting parameters.

Sample	τ_r (fs)	A_1 (%)	τ_1 (ps)	A_2 (%)	τ_2 (ps)	R^2
Octamer (600 nm Exc)	--------	100	1.2 ± 0.1	--------	--------	0.94
Octamer (500 nm Exc)	--------	94	1.4 ± 0.3	6	24 ± 5	0.99
Octamer (400 nm Exc)	--------	90	1.4 ± 0.2	10	24 ± 5	0.99
Heptamer (600 nm Exc)	--------	100	1.3 ± 0.1	--------	--------	0.98
Heptamer (500 nm Exc)	--------	93	2.7 ± 0.2	7	24 ± 5	0.99
Heptamer (400 nm Exc)	--------	86	2.7 ± 0.2	14	24 ± 5	0.99
Hexamer (500 nm Exc)	--------	100	6 ± 1	--------	--------	0.99
Hexamer (400 nm Exc)	--------	86	6 ± 1	14	100 ± 50	0.97
Pentamer (500 nm Exc)	--------	86	11 ± 2	14	80 ± 15	0.97
Pentamer (400 nm Exc)	--------	60	13 ± 2	40	80 ± 15	0.99
Tetramer (500 nm Exc)	--------	100	60 ± 15	--------	--------	0.95
Tetramer (400 nm Exc)	--------	92	60 ± 15	8	Offset	0.99
Trimer (550/775 nm ESA)	280 ± 50	63	1300 ± 200	37	125000 ± 10000	0.98
Trimer (1100 nm ESA)	IRF	77	0.3 ± 0.1	23	Offset	0.99
Dimer (625 nm ESA)	IRF	99.9	115 ± 15	0.1	1.9 ± 0.2ns	0.98

3/ References:

1. Apperloo, J. J., Martineau, C., Van Hal, P. A., Roncali, J. & Janssen, R. A. J. Intra- and intermolecular photoinduced energy and electron transfer between oligothienylenevinylenes and N-methylfulleropyrrolidine. Journal of Physical Chemistry A 106, 21–31 (2002).
